# A comparative study on the governance approaches for biotechnological risks

**DOI:** 10.3389/fbioe.2025.1647204

**Published:** 2025-08-06

**Authors:** Yuexin Wang

**Affiliations:** School of Philosophy, Huazhong University of Science and Technology, Wuhan, China

**Keywords:** emerging biotechnology, biotechnological risks, governance, the laissez-faire approach, the preventive approach, the precautionary approach

## Abstract

The governance approaches for biotechnological risks mainly include the laissez-faire approach, which emphasizes “technology first” but suffers from lagging governance; the preventive approach, which emphasizes “safety first” and uses quantitative risk-benefit analysis as the main method in a single dimension; and the precautionary approach, which also emphasizes “safety first” but has three dimensions of cognition, procedure, and action, and is characterized by forward-looking and proportionality. Emerging biotechnologies are developing rapidly while being filled with a large amount of uncertainty, which increases the complexity of potential biotechnological risks. The precautionary approach, which emphasizes cultivating a cautious awareness and future responsibility in cognition, reversing the burden of proof in procedure, and ensuring that the level of precautionary measures is proportionate to the level of risk in action, is conducive to dealing with various risks including uncertainty risks and meets the needs of biotechnological risks governance, providing a strong theoretical guarantee for the balance between technological progress and social security development.

## 1 Introduction

The term “risk” can be traced back to the Latin word “riscum” during the Renaissance period (Luhmann, 1993). In its early conception, “risk” referred to objective dangers and their probabilities caused by irresistible forces (Ewald, 1993). For such risks, in this context, the governance measures that humans could take were relatively limited, and the effects of risk governance were also rather restricted. With the advancement of human technology and the innovation of systems, the emergence and continuous development of the industrialized world characterized by modernity (Giddens, 1990), “risk” began to refer to the unintended consequences of human actions. Currently, the term “risk” is typically used to denote negative, undesirable, or unfavorable outcomes (Lupton, 2013), or events that may or may not occur but are undesirable (Hansson, 2022).

In this article, the concept of biotechnological risks refers to events that may occur in the field of biotechnology but are undesirable and have negative impacts. It is the sum of biosafety risks and biosecurity risks in the general sense. The objective of biotechnological risks governance is to reduce accidental exposure and release of biomaterials, prevent biotechnology abuse, biological weapons attacks and bioterrorism, thereby curbing risks and reducing them to an acceptable level for humans (Zhou et al., 2019; Abad et al., 2024; Chinchilla et al., 2024). To address and contain risks, three main risk governance approaches have been developed. The first is the laissez-faire approach, which advocates full trust in technology and remedial measures only after problems arise. The second is the preventive approach, which is to advocate that prevention is better than cure, mainly using the method of quantitative risk-benefit analysis to conduct quantitative evaluations of technologies first, and then carry out the governance. The third is the precautionary approach, which advocates prevention before problems occur and careful consideration before action. It is composed of the “heuristic of fear” in the epistemic dimension (The fear referred to here is not the ordinary kind of fear, but rather an active, rational, caring and forward-looking concern), the reversing the burden of proof in the procedural dimension, and proportionate precautionary principle in the action dimension. It should be noted that, firstly, this precautionary approach is different from the one that merely advocates for restrictive measures based on unscientific assumptions of risks and pursues zero risks at all costs; secondly, it is also distinct from the early precautionary approach that only proposes countermeasures for potential severe and irreversible harm while neglecting to offer countermeasures for other types of uncertain risks.

Although the laissez-faire approach has been continuously refined with the development of the times, its lagging nature is not conducive to the governance of biotechnological risks. The preventive approach is still applicable to the governance of known and quantifiable risks at present, but it is difficult to deal with uncertain risks. The foresight provided by the “heuristic of fear” and reversing the burden of proof in the precautionary approach, as well as the proportionality provided by proportionality governance in the precautionary approach, can adapt to the new situation of biotechnology development and is a suitable approach for the governance of biotechnological risks at present.

## 2 The laissez-faire approach

The term “laissez-faire” in French means “let it be” or “let it go”, referring to the government’s minimal intervention in personal and social economic affairs. Its fundamental belief is that when the government does not interfere in the market, businesses and society will thrive (Britannica Money, 2025). It is said that the term originated from the response given by entrepreneurs to Jean-Baptiste Colbert, the finance minister of King Louis XIV of France, when he asked what the government could do for business: “Don’t interfere with us.” With the rise of classical economics influenced by Adam Smith’s “An Inquiry into the Nature and Causes of the Wealth of Nations”, policies based on laissez-faire gained strong support. Later, the application of the laissez-faire approach began to expand from the economic field to political and other fields, suggesting that when individuals pursue their own goals, they will bring about the best results for the society they belong to, and the role of the state is to enforce contracts, maintain order and security, and avoid interfering with individuals’ initiative in pursuing their own goals (Britannica Money, 2025).

### 2.1 The first specific manifestation of the laissez-faire approach: the assimilative capacity principle

The “assimilative capacity” refers to the ability of the natural environment to absorb and neutralize the adverse effects of pollution. The assimilative capacity principle originated from early environmental risk governance. Its typical definition is found in Principle six of the “Report of the United Nations Conference on the Human Environment” (United Nations, 1972): “When the quantity or concentration of toxic substances or other substances and heat discharged exceeds the harmless transformation capacity of the environment, it must be prevented to ensure that the ecosystem is not subject to serious or irreparable damage.” The assimilative capacity principle generally assumes that science and technology can provide regulators with sufficient and necessary information to accurately determine the limit of the environment’s capacity to bear a specific technological disturbance from human input without causing unacceptable harm. Once this capacity is determined, science and technology can provide regulators with sufficient time, opportunity, and technical support to ensure that the impact of new technological disturbances can be borne by the environment. In other words, this principle holds that science can accurately predict environmental risks and provide technological solutions for accurately predicted risks; regulators have enough time to take action after the prediction (McIntyre and Mosedale, 1997).

### 2.2 The second specific manifestation of the laissez-faire approach: the proactionary principle

The proactionary principle is also known as the pro-actionary principle. The core idea of this principle is that progress is vital (More, 2013). The aim of this principle is to combine vigorous technological advance and wise decision-making (More, 2013). Proponents of the proactionary principle hold that emerging science and technology should be considered safe, economically viable, and inherently beneficial until proven otherwise, meaning the burden of proof is on those who want to hinder a specific area of research (Parens et al., 2009), and all proposed measures to restrict new technologies should be carefully examined (More, 2013). Proponents also believe that humans have the ability to continuously evolve to adapt to and remedy any adverse side effects. Therefore, one of the key aspects of the proactionary principle is to propose remedies and restorations after people have suffered risks (More, 2013). That is to say, the proactionary attitude upheld by this principle often leans towards supporting minimal governance, which typically implies the pursuit of self-regulation and self-governance (Parens et al., 2009). Therefore, based on the proactionary principle, researchers and merchants engaged in the trade of related products can carry out their work without restraint until a sufficiently serious risk emerges.

### 2.3 The limitations of the laissez-faire approach

Although the laissez-faire approach can stimulate the creativity and vitality of scientific researchers, thus creating an environment conducive to the growth and development of scientific and technological innovation, “in a world where interests partially overlap and partially conflict, we have every reason to doubt that self-interested behavior at the micro level will spontaneously produce ideal consequences at the macro level” (Denis, 2002). Therefore, the laissez-faire approach will cause a series of negative effects: the regulatory capacity of regulators is weakened; regulatory transparency is reduced; regulatory measures are relaxed or abolished. These negative effects will affect the effectiveness of consumer protection, laborer safety and environmental protection, thereby causing adverse impacts on people’s health and safety, the sustainable development of the ecological environment and social fairness and justice.

Specifically, if the assimilative capacity principle in the laissez-faire approach is followed, the possible outcome could be that “conclusive scientific evidence of the harmful effects of an action or substance comes too late” (Freestone, 1994; Hohmann, 1994), meaning that before any mitigation measures have been developed or applied, the environment may already have become burdened with damaging inputs from which it cannot recover. In other words, the scientific uncertainty that exists in cognition hinders regulators from effectively governing risks based on the assimilative capacity principle. If the proactionary principle in the laissez-faire approach is adopted, the following problems may arise. First, the proactionary principle advocates self-regulation or self-governance, meaning that the trust of regulators and the public can be gained through the self-discipline of the scientific community (Nature Editorial, 2004). However, with the increasing number of business scientists, some of whom are both players and referees, may prioritize their own wealth growth brought by technological innovation over the increase of scientific knowledge or human wellbeing. This may lead to a decline in the self-regulation and self-governance capabilities of the scientific community, thus making it difficult to effectively reduce or mitigate risks. Moreover, it may erode public trust due to the inherent conflicts of interest that commercial technology developers may have when assessing their own technologies. If the public does not believe that technology developers are protecting the public interest, as opposed to their own individual interests, it will likely demand an independent regulatory regime be established that does not rely on self-governance. Second, the proactionary principle emphasizes taking action first, and the risks caused by the action are what humans should accept and bear in order to obtain the dividends of technological progress, and serious risks can be remedied and restored later as human risk-solving capabilities improve. However, for those scientific and technological innovations in the early stages, once they fail, the scope of corrective intervention will be extremely limited, and irreversible consequences cannot be solved by later remedial measures (Giese and von Gleich, 2015).

In conclusion, due to the laissez-faire approach’s overall non-prudent attitude of trusting the positive impacts of technology while neglecting or underestimating its negative effects, it has led to a lag in the governance of biosafety risks under this approach. The lagging laissez-faire approach is unable to effectively deal with the irreversible harm caused by biotechnology, which compels people to seek alternative approaches.

## 3 The preventive approach

The core of the preventive approach is the preventive principle, whose main objective is to take action before foreseeable harm occurs to prevent damage to the environment or reduce and limit actions that may pose potential risks. In this approach, preventive actions are based on quantitative analysis. Compared with the laissez-faire approach, the preventive approach has certain improvements in terms of its forward-looking nature and rationality. However, it may have certain limitations when dealing with risks that are uncertain.

### 3.1 The origin and definition of the preventive principle

The first appearance of the preventive principle can be traced back to the no-harm principle established in the “Trail Smelter Case” (this principle is distinct from the principle of non-maleficence in bioethics). It states that no state shall use or permit the use of its territory in such a manner as to cause substantial damage to the territory, property or persons of another state, and there is clear and convincing evidence of such damage (United Nations, 2006). Since the no-harm principle aims to reconcile the sovereign right of each state to develop its own resources in accordance with its own policies with the fact that such rights are restricted when they cause environmental damage to other states, it is more about preventing cross-border conflict risks than environmental risks. However, the emergence of this principle has already revealed one of the key features of the preventive principle - it targets serious, well-documented damage or harm. The no-harm principle in the “Report of the United Nations Conference on the Human Environment,” also known as the “Stockholm Declaration and Action Plan for the Human Environment,” states that “states have the sovereign right to exploit their own resources in accordance with their own environmental policies, and they also have the responsibility to ensure that activities within their jurisdiction or control do not cause damage to the environment of other states or to areas beyond national jurisdiction” (United Nations, 1972). This principle has endowed the preventive principle with the characteristic of focusing on the prevention of environmental risks.

Later, as “prevention is no longer conceived of from a state-sovereignty perspective” (De Sadeleer, 2020), we can consider that this principle has expanded from mainly being applied to environmental risk prevention among countries to being applicable to the prevention of risks in various entities and fields (including the prevention of biotechnological risks). For instance, the pollution prevention framework, which holds that it is easier to prevent problems than to correct them (U.S. Environmental Protection Agency, 2012) is in line with the preventive principle. The pollution prevention framework uses the risk assessment process to identify hazards (toxicity) (determining if a particular chemical is or is not causally links to particular health effects), conduct dose-response assessment (determining the relation between the magnitude of exposure and the probability of occurrence of the toxicity/hazard effects of concern), carry out exposure assessment (determining the extent of exposure before or after application of regulatory controls), and describe risk characteristics (determining the potential for, and magnitude of, risk to an exposed individual or population nature) (U.S. Environmental Protection Agency, 2012). On this basis, quantitative risk-benefit analysis can be conducted. When that pollution has health consequences for those exposed to it, the harms from that pollution can be calculated quantitatively—at least in part—as a function of those health consequences. Similarly, the cost of pollution control equipment or process changes sufficient to bring pollution down to acceptable levels can also be calculated, making a direct comparison possible (Of course, any actual regulatory decision would have to recognize that the parties benefiting from the process generating the pollution are different from the parties suffering the health consequences, so simply equating costs and benefits would likely not be appropriate—but the point is that a quantitative analysis is possible that can contribute to the overall decision).

In conclusion, the application of the preventive principle is predicated on the existence of relatively clear and convincing evidence of harm. That is to say, the preventive principle is mainly applicable to risks where there is relatively sufficient scientific evidence of a causal relationship between actions and harmful consequences, and where the severity of the risk and/or the probability of risk occurrence are known and quantifiable.

### 3.2 The limitations of the preventive principle

#### 3.2.1 The limitation of the starting point

The preventive principle is based on the starting point that “prevention is better than cure” and “preventive interventions are more effective than later remedial action” (The Lancet Psychiatry Editorial, 2022), emphasizing the prevention of known risks or certain damages. In reasoning based on this starting point, the trigger condition for the preventive principle is well-founded scientific understanding, such as a clear causal relationship between action and consequence being known, or the probability of risk occurrence being quantifiable. This makes the preventive principle less applicable in cases of scientific uncertainty.

#### 3.2.2 The limitation of the objects of governance

The preventive approach targets “risk of environmental harm that is foreseeable and of a certain magnitude” (Duvic-Paoli, 2018), that is, “risks for which causation between an event and damage is demonstrated by irrefutable scientific proof” (De Sadeleer, 2021). That is to say, the risks targeted by the preventive approach are either those that “risks taking the form of a high probability of causing significant transboundary harm” or those that “risks taking the form of a low probability of causing disastrous transboundary harm” (International Law Commission, 2001). It is not difficult to see from the definition of the risks governance by the preventive approach that its aim is to assess the magnitude of harm that risks may be caused and the probability that the risks will materialize (Duvic-Paoli, 2018); or, in other words, to “establish the causal link between the initial event and its adverse effects, to calculate the probability of their occurrence” (De Sadeleer, 2020). Therefore, it can be said that the risks targeted by the preventive approach can be characterized as certain risks. Since certain risks are risks that require the calculation of the probability and severity of their occurrence, such risks are usually known risks and quantifiable risks. Among them, known risks refer to risks where there is scientific evidence proving a causal relationship between an event and damage; quantifiable risks refer to risks where the likelihood and nature of the expected impact have been relatively determined.

The preventive measures for known risks and quantifiable risks are to anticipate harm in advance and prevent its occurrence and spread when the damage may be irreversible or the cost of remediation is extremely high. The typical application scenarios of the preventive principle are the traditional biological industries, as the degree and probability of environmental harm caused by traditional industries are usually relatively clear and known. However, for emerging biotechnologies with significantly increased scientific uncertainties, the preventive principle is difficult to handle with ease.

#### 3.2.3 The limitation of countermeasures

A comprehensive and systematic assessment of all “environmental impacts” of a specific project, that is, all direct and indirect, short-term and long-term, temporary and permanent, accidental or intentional effects on various environmental elements (De Sadeleer, 2020), is often regarded as a prominent feature of the preventive approach. And in accordance with the preventive approach, public authorities should assess whether the cost of their actions would exceed the cost of the damages that might be avoided (De Sadeleer, 2020), and then decide whether to take preventive measures. Therefore, it can be said that the main measures of the preventive approach are to conduct environmental risk assessment or environmental impact assessment around quantitative risk-benefit analysis, so as to obtain relatively clear scientific evidence. Quantitative risk-benefit analysis is a type of risk-benefit analysis (also known as cost-benefit analysis). It is a decision-making support technique that measures the pros and cons in a quantitative way and in numerical form, and it assigns monetary value to all potential outcomes, thereby simplifying multi-dimensional problems into one-dimensional ones (Hansson, 2022). Because risk-benefit analysis is highly malleable, it can be regarded as either quantitative risk-benefit analysis or qualitative risk-benefit analysis (Spielthenner, 2012). Among them, quantitative risk-benefit analysis requires quantifying the results of available alternatives by assigning numerical values to risks and benefits; while qualitative risk-benefit analysis mainly includes identifying threats or opportunities, their likelihood of occurrence, and the potential impacts if they do occur (Safran, 2025).

The prerequisite for conducting a quantitative risk-benefit analysis based on a preventive approach is to have a relatively comprehensive understanding of the potential risks and benefits of technology (all forms of harm and benefits should be as predictable as possible). Once the risks and benefits of a proposed study have been recognized, the subsequent step is to quantify them (Coleman, 2021). Due to the considerable scientific uncertainties in some biotechnologies, these aspects cannot always be effectively quantified and evaluated at present. This situation will affect the scope of the preventive approach in curbing the risks of biotechnologies. Moreover, as the quantitative risk-benefit analysis adopted by the preventive approach unifies and simplifies various values into a single metric (monetary value), it is difficult to fully encompass the diverse values of emerging technologies (Aldred, 2022).

In conclusion, although the preventive approach adheres to “safety first” and is more forward-looking than the laissez-faire approach, due to the numerous limitations mentioned above, it still cannot effectively address the increasing uncertainties of emerging biotechnologies.

## 4 The precautionary approach

The core of the precautionary approach is the precautionary principle, whose main objective is to avoid serious and/or irreversible harm that poses risks to human beings and/or the natural environment. The precautionary approach is similar to the preventive approach in emphasizing safety first, but different from the laissez-faire approach. One of the differences between the precautionary approach and the preventive approach is that the latter is mainly used to deal with known and quantifiable risks, while the former can not only be applied to the governance of known and quantifiable risks, but also to the governance of risks with uncertainties. Another difference between the precautionary approach and the preventive approach is that the latter is a single-dimensional governance approach mainly centered on quantitative risk-benefit analysis, while the former is a multi-dimensional governance approach composed of epistemic dimension, procedural dimension and action dimension. Note that “precautionary” does not necessarily mean without reference to scientific understanding, and that a “proportionate precautionary principle” is proposed below that states that precautionary measures should be proportionate to anticipated risks.

### 4.1 The precautionary principle in the epistemic dimension

The objective of the precautionary principle in the epistemic dimension is to inform us how to perceive risks. A typical example of the precautionary principle in the epistemic dimension is the “heuristic of fear” proposed by the German philosopher Hans Jonas. The fear in this method is not a “approachological” fear (the kind of fear that involuntarily subdues us when facing the object of fear); rather, it is a spiritual attitude that stems from our deliberate education and cultivation of our souls, making us willing to be moved merely by the thought of the possible wellbeing or misfortune of future generations (Jonas, 1984). In other words, based on the fear of the future, human actions will not be rash but cautious, and people will shoulder the responsibility for the world (Jonas, 1987).

The basic connotation of the “heuristic of fear” is that pessimistic predictions are given more weight than optimistic ones (Jonas, 1984), although not to the point of overruling all other considerations. That is to say, one should prioritize predicting the possibilities that are terrifying in the future, thereby stimulating the awareness to correct one’s actions and minimize disasters (Fang, 2004). The “heuristic of fear” brings two inspirations. The first is that although the things to be feared have never occurred and there may be no similar experiences in the past or present, we have the responsibility to replace the “experienced malum” with the “creatively imagined malum” and through reason and imagination, strive to envision the long-term impact of technology (Jonas, 1984). The second is that although the imagined distant risks are not directed at us personally and thus do not naturally and spontaneously evoke fear like imminent risks, we have the responsibility to evoke a premonition proportionate to the envisioned things and cultivate an attitude that can generate fear when facing merely speculative and distant predictions concerning the fate of humanity, thereby making our senses sensitive to such stimuli in advance (Jonas, 1984).

One of the reasons why we need the “heuristic of fear” in the epistemic dimension is that the risks of modern technology are not yet fully known and understood, which leads to our lack of clarity about the reasons for protection, the specific objects we should protect, and the value of the things we should cherish; only when we realize that something is in danger do we truly understand its value (Jonas, 1984). That is to say, we need to indirectly prompt the discovery of the potential value of future things through the emotional cognition of fear.

The second reason is that the power of modern technology has surpassed all known and even previously imagined human power and is growing at an accelerating pace; when facing modern technology, due to the insufficiency of the knowledge required for prediction, if we make inferences based on the currently available data, there may be deficiencies in the certainty and completeness of the prediction. Considering the severity of the risks of modern technology, we must be vigilant (Jonas, 1984). That is to say, in traditional ethical ideas, fear is regarded as an emotion that hinders thinking, but in Jonas’ “heuristic of fear”, fear is endowed with an enlightening function and becomes the basis for taking responsibility. Fear prompts people to pay attention to any issue with a negative future prospect and serves as a warning signal (Pugacheva, 2020). And it cannot be ignored that fear is a special emotion with rational connotations, and it has the power to unify the rational and emotional dimensions of humanity (Tibaldeo, 2015), and this power is precisely manifested through the sense of responsibility that fear brings, which can balance reason and emotion. Furthermore, fear is the primary obligation of Jonas’ ethics of responsibility, which prompts people to take action in the new era’s technology to discover the value of prediction and reflect on their own destiny, etc. (Nodari and Mezzomo, 2024). This also reflects the rational side of fear.

The third reason is that the fate of future humanity or the fate of the Earth neither involves us nor anyone else who is connected to us by love or coexistence, so the distant future itself does not evoke a strong sense of fear in us (Jonas, 1984). However, when dealing with global issues, we have a greater need to be cautious and responsible. Therefore, we need to re-examine the cultivation of researchers’ sense of responsibility in the field of scientific research, stimulate their fear, and let fear prompt researchers to consider future generations (Pugacheva, 2020).

In conclusion, the fear in the “heuristic of fear” will direct emotions towards cognition, and from cognition towards responsibility (Teles and Cabral, 2021). This method opposes putting humans in danger and emphasizes the importance of moderation and prudent action (Fang, 2004).

### 4.2 The precautionary principle in the procedural dimension

The precautionary principle in the procedural dimension does not directly stipulate the action plan to be taken, but rather points out the requirements that can be adopted in the process of applying the precautionary principle. Reversing the burden of proof is a typical manifestation of the precautionary principle in the procedural dimension.

Reversing the burden of proof is not something that emerged out of thin air; its appearance has historical reasons. The concept of the “burden of proof” emerged initially, which indicates that all parties have the responsibility to prove the claims they put forward (Taylor, 2023). That is to say, “in the International Court of Justice, all parties to the dispute have the obligation to prove their claims to the satisfaction of the court and in accordance with the rules recognized by the court” (Kazazi, 1996). In practice, this concept is generally regarded as the party making a claim bearing the burden of proof for its claim (Lasok, 1994; Amerasinghe, 2005), and fulfilling the burden of proof through evidence, presumptions or judicial inferences (Foster, 2011). This means that either the objector (i.e., the plaintiff) or the respondent (i.e., the defendant) can become the claimant who bears the burden of proof. However, in the field of biotechnology, the concept of “burden of proof” has transformed into that of the “burden of proof in the presumption of innocence”. This means that when the concept of “burden of proof” is applied in the field of biotechnology, those who believe that a certain biotechnology has potential risks (i.e., the skeptics and objectors of biotechnology) are often regarded as having the responsibility to provide scientific evidence and prove their point; while those who believe that a certain biotechnology has no potential risks (i.e., the biotechnology advocates and practitioners who originally needed to defend themselves) are often tacitly assumed to have no responsibility to provide scientific evidence and prove their point (no need to defend). In response to the potential liability crisis and adverse effects of the concept of the “burden of proof in the presumption of innocence”, the concept of “reversing the burden of proof” emerged. Contrary to the concept of the “burden of proof in the presumption of innocence”, the concept of Reversing the burden of proof shifts the burden of proof from the “plaintiff” to the “defendant” (from the biotechnology skeptics to the biotechnology advocates and practitioners), and shift from the presumption of the defendant being innocent to the presumption of the defendant being guilty.

One of the reasons why “reversing the burden of proof” is superior to “burden of proof in the presumption of innocence” in the context of emerging biotechnology is that the public finds it difficult to conduct adequate monitoring, investigation and risk assessment of potentially risky actions, especially the rapidly developing emerging biotechnology. Therefore, the “burden of proof in the presumption of innocence”, which requires the public to provide scientific evidence, cannot be realized. It is more appropriate for the burden of proof to be shouldered by researchers and advocates who have a better understanding of the technology. The second reason is that the lag and limitations in the public’s understanding of emerging technologies may affect their response speed to risks. If governance actions can only be taken after the evidence of harm caused by actions is fully confirmed by the public, it may lead to an irreversible predicament where the harm cannot be mitigated. (The government, acting on behalf of the public, can conduct research related to risk assessment and risk management in selected topics, as the U.S. Department of Agriculture did in its “Biotechnology Risk Assessment Research Grants (BRAG)” (U.S. Department of Agriculture, National Institute of Food and Agriculture, 2025). However, it would likely not be feasible for government to maintain a standing research capacity to anticipate and fund risk assessment in all areas of potentially risky emerging technology).

### 4.3 The precautionary principle in the action dimension

The precautionary principle in the action dimension tells us which action plan to choose in a specific situation. A typical example is the proportionate precautionary principle (the precautionary principle with proportionality).

In the dimension of action, the precautionary principle in its early stage (the form of the precautionary principle in its early stage of development) has already begun to attach importance to safety and the prevention of risks (which makes it somewhat similar to the preventive principle); the objects of governance under the early precautionary principle have started to focus on potential risks that cannot be fully assessed at present (which distinguishes it from the preventive principle); in addition, the early precautionary principle does not clearly reflect proportionality (which differentiates it from the later “proportionate precautionary principle”). For instance, the “the Prevention of Environmental Damage through the Avoidance and Gradual Reduction of Hazardous Substances” (Leitlinien der Bundesregierung zur Umweltvorsorge durch Vermeidung und stufenweise Verminderung von Schadstoffen) of Germany, which is the federal government’s guideline, emphasizes the precautionary principle (referred to as “Vorsorgeprinzip” in German): to prevent potential environmental hazards and risks that cannot be fully assessed at present; for substances that are regarded as problematic due to their impact on humans and the environment during testing, their uses and application methods should be restricted, or even prohibited from entering the circulation field (Deutscher, 1986). The later emerging category of the precautionary principle began to show signs of embodying proportionality. For example, the “Rio Declaration on Environment and Development” states: “In order to protect the environment, the precautionary approach shall be widely applied by States according to their capabilities, and where there are threats of serious or irreversible damage, lack of full scientific certainty shall not be used as a reason for postponing cost-effective measures to prevent environmental degradation.” (The United Nations Conference on Environment and Development, 1992). As the precautionary principle here mentioned that the object of precautionary measures should be serious or irreversible threats and that the measures should be cost-effective, although it does not explicitly mention proportionality, it has actually begun to show a trend that precautionary measures should be proportionate to risks (though it still has a considerable distance from the true “proportionate precautionary principle”).

Per Sandin’s four-dimensional precautionary principle is an early typical example of the proportionate precautionary principle. This principle emphasizes that if there is a threat, which is uncertain, then some kind of action is mandatory (Sandin, 1999; Sandin, 2006). Sandin believes that the analysis of the risks of specific actions should be based on four dimensions: threat, uncertainty, action and command (Sandin, 1999; Sandin, 2006). In the threat dimension, the severity, reversibility and preventability of the threat should be analyzed respectively; in the uncertainty dimension, attention should be paid to the accuracy of the risks disclosed by scientific evidence, the lower the accuracy, the higher the uncertainty, and the higher the accuracy, the lower the uncertainty; in the command dimension, instructions of higher or lower compulsion can be taken according to the threat and uncertainty of the risk; in the dimension of action, strict or loose actions can be taken according to the threat and uncertainty of the risk (Sandin, 1999; Sandin, 2006) (Since the dimensions of action and command mentioned by Sanding are not always clearly distinguishable in all circumstances, the subsequent proportionate precautionary principle often combines the two).

Arie Trouwborst’s three-dimensional precautionary principle (it also known as the “precautionary tripod”) is another early example of the proportionate precautionary principle. This principle holds that the threat of harm, uncertainty, and action are the three dimensions of the precautionary principle (Trouwborst, 2006). In the dimension of the threat of harm, the threshold of the severity of the harm is the decisive factor in determining whether people should take precautionary measures (only significant, severe, and irreversible threats can trigger precautionary measures, while insignificant and trivial threats cannot); in the dimension of uncertainty, attention should be paid to the level of uncertainty brought about by the complexity and variability of objective things, and risks can be classified into quantifiable risks where both the severity and probability of occurrence can be estimated and expressed in numbers, uncertain risks where only the severity can be quantified, and ignorance risks where neither the probability of occurrence nor the severity can be quantified; in the dimension of action, proportionality should be the basis for action (Trouwborst, 2006). For risks that will not cause adverse effects or have insignificant harm, no action is needed; for risks that may cause serious harm, people have the right to take action; and for risks that have a high probability of causing serious and/or irreversible harm, people have the duty to take action (Trouwborst, 2006).

Jonathan Birch’s “precautionary framework for managing major risks” is a typical recent development of the proportionate precautionary principle. This principle holds that “when there is an urgent and credible threat of grave harm, proportionate precautions should be taken” (Birch, 2024). In this principle, the gravity of the risk (harms are sufficiently grave) is an important prerequisite for people’s obligation to take precautionary measures; the urgency of the risk (some serious risks are urgent) is an important basis for people’s obligation to take immediate action rather than waiting for the risk to further develop; the credibility of the risk requires that the evidence provided by people for the severity and urgency of risks, even if it does not have to be conclusive (does not have to be certain, highly probable or highly confident), still needs to have sufficient credibility; and the action proportionate to the risk should be just enough without being excessive (Birch, 2024). To ensure that the precautionary measures and risks are indeed proportionate, Birch proposed the “PARC” test standard to examine proportionality through permissibility-in-principle, adequacy, reasonable necessity, and consistency. Among them, permissibility-in-principle requires that the measures proposed for the identified risks should conform to the common values of people; adequacy requires that the proportionate response to the risk must be strong enough (either to reduce the risk to an acceptable level or, if the former is not achievable, to achieve the greatest possible risk reduction among all feasible options in principle); reasonable necessity requires that precautionary measures should not impose harms or costs that exceed what is reasonably necessary to achieve adequacy; consistency requires that the precautionary measures taken to avoid a certain risk should not trigger new risks of a severity that would cause people to attempt to revoke the initial precautionary measures (Birch, 2024). It can be said that the proportionality feature of the precautionary principle prevents the arbitrariness of people in applying precautionary measures, thereby facilitating the maximization of the advantages of the precautionary principle. The test of the proportionality of the precautionary principle further ensures the full play of its superiority.

It should be particularly noted that the concept of proportionality not only implies that current actions are proportionate to current risks, but also implies a mandate for constant reevaluation and adaptive policymaking. That is to say, if there is new information and evidence about the risks of biotechnology and the policies formulated initially are no longer proportionate, then proportionate decisions need to be made anew and proportionate actions updated. However, the increased unpredictability of the policy environment resulting from such policy changes may become a hidden danger that hinders technological innovation. That is to say, if specific precautionary measures are constantly updated, it may cause confusion and concerns among relevant entities, making it difficult for them to carry out long-term research and investment. However, I believe that although this kind of doubt has some grounds, it can be refuted. I believe that the changes in precautionary measures that adapt to technological development and the changes of technological risks are not necessarily a comprehensive changes, but are more likely to be partial changes. Only when a new development in a certain technology greatly overturns its original foundation is it more likely that the adjustment of proportionate precautionary measures will be comprehensive. Since major self-disruptive technological changes do not always occur at a high frequency, and since the partial technological development that occurs frequently and the proportionate precautionary measures do not have a significant and far-reaching impact on technology developers and investors, they will not hinder the reasonable and normal innovation of technology. Moreover, I believe that proportionality contains necessity. That is to say, when precautionary measures are proportionate to the risks, changes to these measures and policies are necessary rather than superfluous for curbing the risks. Perhaps these changes in measures and policies have to some extent increased the unpredictability of the policy environment, but to a greater extent they have provided certainty for the safe development of technology and the enhancement of human wellbeing.

In summary, the “heuristic of fear” in the epistemic dimension of the precautionary principle makes people have a sense of foreboding before potential risks cause serious harm, and the “reversing the burden of proof” in the procedural dimension of the precautionary principle requires researchers to provide sufficient safety evidence before the application and marketing of technology. Both can reflect the forward-looking characteristic of the precautionary principle. The various versions of the precautionary principle in the action dimension all emphasize that precautionary measures should be proportionate to the degree of risk, which can reflect the proportionality of the precautionary principle. It is precisely the characteristics of forward-looking and proportionality that make the precautionary approach, which is centered on the precautionary principle, more adaptable to the needs of the times for technological development and the characteristics of risks of the times compared with the laissez-faire approach and the preventive approach.

## 5 Emerging biotechnology and the precautionary approach

Synthetic biology is a cutting-edge field of biotechnology (it can be regarded as a typical emerging biotechnology). With the ultimate goal of enhancing human scientific understanding and creating things that can improve human wellbeing, synthetic biology aims specifically at designing and creating non-naturally occurring biological components, devices and systems, as well as redesigning and modifying existing ones. It employs gene editing, DNA assembly and other techniques as its main technological means, and is an emerging science and technology that blends physics engineering and genetic engineering (Holm, 2012; Hunter, 2013; Häyry, 2017). Synthetic biology not only seeks to change biological components, devices and systems, but more importantly, makes it possible to create synthetic biological components, devices and systems that can increase human wellbeing. Targeted drugs, new vaccines, clean energy, new high-quality crops and foods, etc. are all potential applications in this emerging technological field.

Despite its promising prospects, synthetic biology has also raised concerns about the risks it may pose (Presidential Commission for the Study of Bioethical Issues, 2010). For instance, gene therapy holds great promise in clinical treatment and represents a significant achievement of synthetic biology in the medical field; however, germline gene therapy within gene therapy, which involves manipulating human embryos and the modified genes can be inherited, poses significant risks (Cui, et al., 2018). The design and modification of the human germline may cause unforeseen harm to future generations (Baltimore, et al., 2015); it may also increase the risk of other diseases by accident when using gene mutations to reduce the risk of certain diseases, thereby making the recipients and their descendants vulnerable to future health threats (Koplin, et al., 2020). Besides gene therapy, unregulated laboratory experiments may lead to the accidental release of synthetic organisms (such as artificial bacteria, artificial pathogen), exposing potential contacts to incalculable risks.

The latest concern about the risks of synthetic organisms is scientists’ worry about the threat of mirror life. Mirror life is a type of synthetic organism that is entirely composed of molecules that are mirror images, or opposite chirality, of the corresponding molecules found in a normal organism (Wizevich, 2024). The two technical approaches to creating a mirror organisms are top-down and bottom-up. In the top-down approach, scientists would introduce new functions into a natural-chirality cell that would gradually transform it into a mirror cell. In the bottom-up approach, scientists would manufacture cellular components having opposite chirality from their normal life counterparts and then assemble these into a mirror synthetic cell (Adamala, et al., 2024a). People believed that mirror life might interfere with the normal immune recognition of human, animal and plant species, evade immune mechanisms and cause fatal infections; mirror life may multiply rapidly and spread widely across multiple ecosystems, becoming invasive species that affect a large number of plant and animal species and thereby causing irreversible damage to ecosystems (Adamala, et al., 2024b).

It can be said that the technical uncertainties of synthetic biology (the technology is still in its development stage, and its long-term effects and potential risks have not been fully clarified), as well as the uncertainties regarding the specific manifestations, severity, and probability of occurrence of risks in synthetic biology (Matthews, et al., 2025), have led to the uncertainty of governance approaches (the specific governance policies that various countries and regions choose to formulate based on which approach).

Due to the lagging nature of the laissez-faire approach in risk governance and the limited scope of the preventive approach in addressing risks, the precautionary approach seems more competent in managing the biosafety risks associated with emerging biotechnologies, such as synthetic biology. However, the choice of the precautionary approach is not merely based on elimination but also on a specific analysis of the precautionary approach and synthetic biology. Firstly, the precautionary approach starts from the principles of “in dubio pro natura” (If in doubt, decide in favor of the environment) (Ahteensuu and Sandin, 2012) and “better safe than sorry”, which require proactive actions to avoid potential serious or irreversible harm in the face of uncertainty. Based on this starting point, prospective governance can be carried out as long as there are reasonable grounds for concern (Trouwborst, 2009). This enables those following the precautionary approach to take early actions and prevent the situation where the rapid development of synthetic biology outpaces governance and becomes incompatible. Secondly, the precautionary approach has a wide range of application. It can not only conduct risk assessment and governance for known and quantifiable risks but also address potential threats lacking scientific certainty. This allows synthetic biology, characterized by “creation for knowledge”, where scientific evidence for safety is insufficient and risk assessment cannot be completed, but where governance measures are urgently needed to deal with potential risks, to develop robustly. Finally, the main measures of action under the precautionary approach revolve around proportionality. Given the diversity of research fields and application areas in synthetic biology, which further complicates the biosafety risks of synthetic biology, only by adhering to the proportionate precautionary principle and ensuring that the action plan is proportionate to the gravity, urgency, and credibility of the risks in specific circumstances, can risks be effectively managed while controlling the cost of actions, avoiding the waste of governance efforts and resources, and preventing overly strict measures from hindering the benign development of technology and social progress.

## 6 Conclusion and expectation

Among the various governance approaches to biosafety risks, the laissez-faire approach, which emerged earliest, is the least advisable. This is because it has a certain degree of governance lag, thus failing to effectively prevent the occurrence of potential threats. Unlike the laissez-faire approach that advocates “technology first”, the preventive approach that emerged later, which advocates “safety first”, is somewhat effective in dealing with known and quantifiable risks. However, most of the emerging biotechnologies represented by synthetic biology nowadays present increasing uncertainties. This limits the governance scope of the preventive approach, which overly relies on risk-benefit analysis methods and has a single-dimensional feature. The precautionary approach, which also advocates “safety first” but has multi-dimensional features and continuously improves itself along with technological development, can enhance people’s caution and sense of responsibility towards future generations in the epistemic dimension; transfer the burden of proof to researchers who are more familiar with new biotechnologies in the procedural dimension, enabling researchers to conduct research and innovation responsibly; and make precautionary measures proportionate to risks in the action dimension, thus unifying the steady progress of technology with the benign development of society.

It is not difficult to see that the precautionary approach, which can reflect forward-looking and proportionality, is the most reasonable and appropriate for emerging biotechnologies such as synthetic biology. After clarifying that the precautionary approach is the main development direction of biotechnological risks governance in the present and future, issues such as how to identify, predict and assess the gravity, urgency and credibility of different biotechnological risks; how to concretize precautionary measures in specific biotechnology research and application fields; and how to coordinate the dominant positions of actors such as the government, technology developers and the public in the application of the precautionary approach, need to be further considered.
